# BikeMaps.org: A Global Tool for Collision and Near Miss Mapping

**DOI:** 10.3389/fpubh.2015.00053

**Published:** 2015-03-30

**Authors:** Trisalyn A. Nelson, Taylor Denouden, Benjamin Jestico, Karen Laberee, Meghan Winters

**Affiliations:** ^1^Spatial Pattern Analysis and Research (SPAR) Laboratory, Department of Geography, University of Victoria, Victoria, BC, Canada; ^2^Faculty of Health Sciences, Center for Hip Health and Mobility, Vancouver Coastal Health Research Institute, Simon Fraser University, Vancouver, BC, Canada

**Keywords:** active transportation, bicycling safety, near miss, citizen science, cycling safety surveillance

## Abstract

There are many public health benefits to cycling, such as chronic disease reduction and improved air quality. Real and perceived concerns about safety are primary barriers to new ridership. Due to limited forums for official reporting of cycling incidents, lack of comprehensive data is limiting our ability to study cycling safety and conduct surveillance. Our goal is to introduce BikeMaps.org, a new website developed by the authors for crowd-source mapping of cycling collisions and near misses. BikeMaps.org is a global mapping system that allows citizens to map locations of cycling incidents and report on the nature of the event. Attributes collected are designed for spatial modeling research on predictors of safety and risk, and to aid surveillance and planning. Released in October 2014, within 2 months the website had more than 14,000 visitors and mapping in 14 countries. Collisions represent 38% of reports (134/356) and near misses 62% (222/356). In our pilot city, Victoria, Canada, citizens mapped data equivalent to about 1 year of official cycling collision reports within 2 months via BikeMaps.org. Using report completeness as an indicator, early reports indicate that data are of high quality with 50% being fully attributed and another 10% having only one missing attribute. We are advancing this technology, with the development of a mobile App, improved data visualization, real-time altering of hazard reports, and automated open-source tools for data sharing. Researchers and citizens interested in utilizing the BikeMaps.org technology can get involved by encouraging citizen mapping in their region.

## Introduction

Cycling has many health benefits (1, 2) and cycling promotion supports better population health. A primary barrier to increased ridership is the risk, both real and perceived, of incurring substantial injury (3, 4). In past decades, overall levels of ridership have increased in North America and cyclist fatalities have declined (5). However, cyclists are still at a greater injury risk than automobile drivers, and there is substantial spatial variation and socioeconomic inequality in cycling rates and safety (5).

Data and studies on cycling safety typically rely on data for crashes between cyclists and motor vehicles (6–8), which are often reported through vehicle insurance claims and/or when police are called to a vehicle crash event. However, cycling safety concerns go beyond motor vehicles incidents. In a study of injured adult cyclists, treated in emergency departments, only 34% of incidents were collisions with motor vehicles and another 14% were a result of avoidance of a motor vehicle (9). Significant injury risk present on multi-use paths, away from motor vehicles (10), and may involve falls or collisions with infrastructure, pedestrians, cyclists, or animals. Importantly, cyclists perceive multi-use (pedestrian and cycling) pathways as safer than they are, based on observed risk (4). The lack of complete datasets on cycling incidents is limiting researchers’ ability to study cycling safety. More comprehensive data are required to assess safety and risk, overcome the gap between real and perceived safety issues, monitor progress in decision-making aimed at improving traffic safety, and identify priority locations for improved traffic management.

Data on near miss incidents are not reported by standard traffic data collection systems, but are a critical aspect of safety management (11). Near miss data have the potential to assist in early detection of high-risk areas and to mitigate both real and perceived safety issues, thereby enabling increased ridership (12). When compared to the number of human errors or near miss incidents, a crash is a relatively rare event. Thus, collecting near miss data allows larger data sets to be generated and enables earlier detection of problematic areas (13), and supports robust statistical analysis.

Volunteer geographic information (VGI), sometimes referred to as geo-crowdsourcing, is a data collected by ordinary citizens through digital mapping, typically, via a web-interface (14). VGI offers an innovative digital technology approach to enriching available data for a wide range of research and planning applications. VGI is emerging as an important tool for health research and practice (15, 16). For instance, Robertson et al. (17) used VGI provided by veterinarians to conduct surveillance of zoonotic disease in Sri Lanka. VGI offers a powerful approach to generating more comprehensive, map-based data on cycling crashes and exposure [i.e., Ref. (3)]. In the area of active transportation, Apps like CycleTracks^1^ (accessed February 1, 2014) and Brisk Cycle^2^ (accessed February 1, 2014) are examples of tools that support collection of cycling specific VGI.

Our goal is to introduce a new tool for collecting data on bicycle safety and risk, developed by the authors. BikeMaps.org is a global web-mapping system that allows citizens to map cycling collisions and near misses, and to identify the location of hazards and thefts. Here, we focus on the functionality associated with mapping cycling collisions and near misses. To introduce this tool, we begin by providing details of the BikeMaps.org technology and outlining website functionality. We then quantify the information content of early data submissions. In the Section “Discussion,” we highlight new opportunities related to BikeMaps.org, highlight technology developments that are underway, and outline how researchers and planners can get involved.

## BikeMaps.org

**Figure 1:**
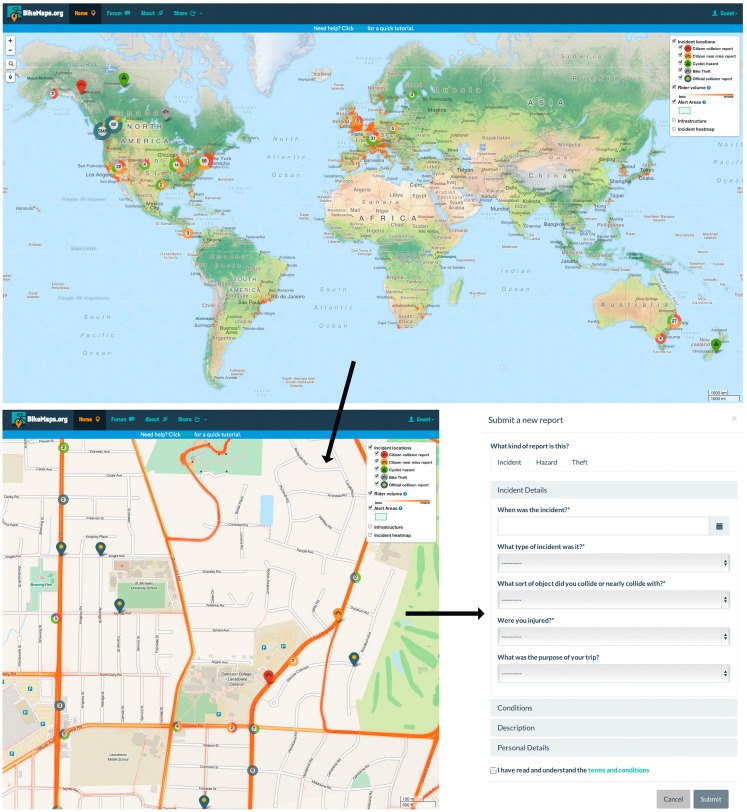
**BikeMaps.org is a global tool for citizen mapping of cycling collisions, near misses, hazards, and thefts**. The upper panel shows the website and global map. The lower panel – left is a close up of the map and the view typically used by the citizen mapper when adding data. The lower – right is an example of the table used to collect attribute data on each cycling incident.

BikeMaps.org is a tool for mapping bike collisions and near misses (Figure 1) and is built with free and open-source tools. The website is welcoming; the citizen mapper sees a GoogleMaps-like interface, although the map technology used is Leaflet, a JavaScript mapping library that can retrieve and render image-like “map tiles” from a map tile server, as well as display point, polyline, polygon, and popup features. The backend database system is PostgreSQL, a database that accommodates efficient storage and querying of spatial data. The website front-end HTML templates use additional open-source JavaScript and CSS packages to provide professional styling, dynamic user interaction, and containers for rendering map content. The website employs the Django web framework to control the retrieval and submission of data between the backend database and front-end templates.

Citizen mappers identify the location of their cycling incident by clicking a “submit new point” button and adding the location on the map where the incident occurred. They then report details of collisions and near misses on a digital form through pull-down options. All reports are anonymous. The attributes captured through the pull-down menus are designed to enable research on important determinants of cycling injury, based on research by Teschke et al. (10). There are three categories of attributes: incident details (Table 1), conditions (Table 2), and personal details (Table 3), with a balance of required and optional questions to manage citizen mapper burden. Most incident details are required fields; citizen mappers are invited to answer questions on when the collision or near miss occurred, the type of object involved, and whether the object was stationary or moving. A question on injury severity is also required, and optionally the citizen mapper can provide details on their cycling trip purpose. The questions around conditions at the location and time of the collision or near miss are optional. The questions query road condition, sight lines, presence of parked cars, type of road, bike infrastructure, use of lighting, terrain characteristics, direction of travel, and traffic flow of cyclist (turning or straight). Personal details are also optional and include details on the birthdate (month and year) of rider (for future, anonymous linking with emergency room health outcomes), gender, cycling frequency, helmet use, and intoxication.

**Table 1:** **Attributes collected on crash or near miss details**.

Crash or near miss details	Responses	%
When was the incident?^a^
Select date and time	356	100.0
What type of incident was it?^a^
Collision with stationary object or vehicle	11	3.1
Collision with moving object or vehicle	101	28.4
Near collision with stationary object or vehicle	11	3.1
Near miss with a moving object or vehicle	211	59.3
Lost control and fell	22	6.2
What sort of object did you collide or nearly collide with?
Vehicle – head on	28	7.9
Vehicle – side impact	185	52.0
Vehicle – angle impact	65	18.3
Vehicle – rear end	20	5.6
Vehicle – open vehicle door	9	2.5
Another cyclist	13	3.7
Pedestrian	5	1.4
Animal	3	0.8
Infrastructure – curb	2	0.6
Infrastructure – train tracks	2	0.6
Infrastructure – pothole	4	1.1
Infrastructure – lane divider	1	0.3
Infrastructure – sign/post	1	0.3
Infrastructure – roadway	5	1.4
Infrastructure – other (please describe)	13	3.7
Were you injured?^a^
Medical treatment not required	44	12.4
Saw a family doctor	20	5.6
Visited the hospital emergency department	38	10.7
Overnight stay in hospital	10	2.8
No injury	244	68.5
What was the purpose of your trip?
To/from work or school	237	66.6
Exercise or recreation	64	18.0
Social reason (e.g., movies, visit friends)	26	7.3
Personal business	22	6.2
During work	2	0.6
No response	5	1.4

*^a^Indicates a required field*.

**Table 2:** **Attributes collected on conditions when the near miss took place**.

Conditions	Responses	%
What were the road conditions?
Dry	230	64.6
Wet	53	14.9
Loose sand, gravel, or dirt	2	0.6
Icy	0	0.0
Snowy	0	0.0
I do not remember	4	1.1
No response	67	18.8
How were the sight lines?
No obstructions	239	67.1
View obstructed	26	7.3
Glare or reflection	10	2.8
Obstruction on road	4	1.1
Do not remember	7	2.0
No response	70	19.7
Were there cars parked on the roadside?
Yes	61	17.1
No	214	60.1
I do not know	13	3.7
No response	68	19.1
Where were you riding your bike?
Busy street – on a painted bike lane	82	23.0
Busy street – on road with no bike facilities	104	29.2
Quiet street – on a painted bike lane	14	3.9
Quiet street – on road with no bike facilities	50	14.0
On a physically separated bike lane	13	3.7
On a mixed use trail	23	6.5
On the sidewalk	4	1.1
I do not remember	0	0.0
No response	66	18.5
Were you using bike lights?
No lights	143	40.2
Front and back lights	122	34.3
Front lights only	6	1.7
Back lights only	14	3.9
I do not remember	4	1.1
No response	67	18.8
What as the terrain like?
Uphill	31	8.7
Downhill	64	18.0
Flat	195	54.8
I do not remember	1	0.3
No response	65	18.3
What direction were you heading?
N	47	13.2
NE	10	2.8
NW	10	2.8
S	56	15.7
SE	10	2.8
SW	8	2.2
E	75	21.1
W	65	18.3
I do not know	7	2.0
No response	68	19.1
How were you moving?
Heading straight	259	72.8
Turning left	23	6.5
Turning right	12	3.4
I do not remember	2	0.6
No response	60	16.9

**Table 3:** **Attributes collected on personal details of the reporting cyclist**.

Personal details	Responses	%
What is your year and month of birth?
Select year and month	170	47.8
No response	186	52.2
Please select your sex
Male	149	41.9
Female	61	17.1
Other	1	0.3
No response	145	40.7
Do you bike at least once a week?
Yes	241	67.7
No	2	0.6
I do not know	1	0.3
No response	112	31.5
Were you wearing a helmet?
Yes	237	66.6
No	10	2.8
I do not know	0	0.0
No response	109	30.6
Were you intoxicated?
Yes	2	0.6
No	239	67.1
I do not know	1	0.3
No response	114	32.0

There are additional functions and visualizations on BikeMaps.org designed to enhance data communication and community engagement. Ridership data are essential for characterizing exposure (1). Without ridership data, collision and near miss hot spots may simply represent rider hot spots (18). We have plans to collect ridership data through a mobile application that is under development. Currently, we provide ridership data available from Strava.com, as a visualization backdrop on the website. Strava.com publishes their ridership data as a map tile dataset, and to our knowledge it is the only publically available data for ridership globally. However, Strava.com best represents the routes of recreational riders and the number of users varies regionally.

We have also added base map data for cycling infrastructure, for our pilot case study in Victoria, British Columbia. Infrastructure is mapped by three categories: protected bike lanes, bike lane, and other cycling routes, similar to what is used by in Bike Score^3^ or Google Maps cycling routes. At present, we add bike infrastructure on a region by region basis, and are developing a framework for more automated submission of cycling infrastructure geographic information system (GIS) files.

On the BikeMaps.org website, each type of incident has a unique marker color. Official data sources, such as police crash reports, can also be incorporated and have a unique symbol. For example, in British Columbia, Canada, we have included data provided by the provincial insurance provider [Insurance Corporation of British Columbia (ICBC)] on collisions including cyclists. The website symbology changes depending on scale: as a user zooms in on the map, a marker appears at the collision or near miss location and certain incident details appear if the user clicks on the marker. As the user zooms out to a larger area, the incident markers aggregate to show the total number of events that have been mapped. For aggregated mapping of incidents, a symbol similar to a pie chart is used to denote the number of each type of incident that has been mapped. Regardless of the scale the data are viewed at, general trends can be visualized through a heat map tool, available in the legend.

Furthermore, BikeMaps.org can generate summary reports. Citizen mappers, researchers, or planners can create a login and define their riding or study area via a polygon. They can then accessBikeMaps.org/stats page to monitor monthly reports on what has been mapped in their riding area. The “stats” page includes a map of the riding area with the frequency of collisions and near misses added. The bottom panel of the “stats” page is used to provide messages to citizens such as social media links for BikeMaps.org, updates on global mapping, and safety messages. For example, currently we include a graphic that demonstrates that cycling is safe through comparison with other travel modes.

## Early Results of Data Submissions

On October 6, 2014, we launched the BikeMaps.org website through a media release. We also emailed bike groups and started a twitter account. Locally, in Victoria, British Columbia, Canada, newspapers, radio, and television stations reported on the project. Globally, social media has been the primary source of promotion.

**Figure 2:**
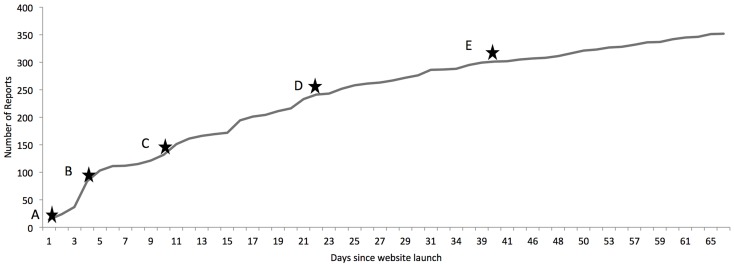
**Number of citizen reports since website launch**. (A) Date of initial press release and local radio interview. (B) Date of local newspaper article. (C) Date of regional TV news report. (D) Date of local newspaper article paper. (E) Date of radio interview.

BikeMaps.org had 14,309 website visitors and 356 reports of collisions (including falls) and near misses within the first 2 months (October 6 to December 6 2014). The number of submissions per day is shown in Figure 2. Days with the highest number of reports were typically associated with newspaper articles about the website. Collisions represent 38% of reports (134/356) and near misses 62% (222/356). Additional sites were mapped in association with hazards and thefts, but are not discussed as part of this paper as the hazard and theft reporting functions are still under development.

Incidents were mapped in 14 countries: Australia, Belgium, Canada, Czech Republic, Sweden, France, Costa Rica, Germany, the Netherlands, New Zealand, Spain, Switzerland, United Kingdom, and the United States. The initial launch targeted citizens in Victoria, Canada and 45% (160) of incidents reported were for Victoria and 75% (266) of reports were within Canada.

**Figure 3:**
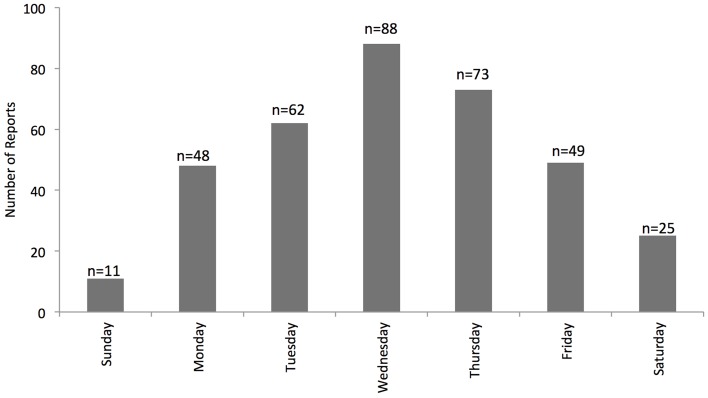
**Day of week trends for citizen reports of collisions and near misses**.

The citizen-mapped incidents were mainly recent reports, with 77% being collisions or near misses that occurred in 2014, 16% from 2013, and 7% from incidents before 2013. The earliest possible submission dates are 10-year prior to the date of reporting. There were strong day of week trends in when the incident occurred (Figure 3). Incidents were most common mid-week, with 25% of incidents occurring on Wednesday and 63% occurring between Tuesday and Thursday. Only 10% of collisions and near misses occurred on the weekend.

In this early stage, we can see that BikeMaps.org makes a significant contribution to the need for more comprehensive data collection. For example, in Victoria, the only prior source of geo-located cycling incident data was cyclist-involved motor vehicle crashes reported to the provincial vehicle insurance carrier (ICBC). The ICBC data include between 119 and 140 reports per year from 2009 to 2013. In comparison, for Victoria, there were 160 citizen reports captured through BikeMaps.org within these first 2 months. Thus, the contribution from citizen mappers to BikeMaps.org in the first 2 months provided as many incident points as might be expected in a year from the existing available cycling incident data from ICBC.

Another early indication of the quality of data generated from BikeMaps.org is the completeness of attributes provided. Fifty percent of data are fully attributed, with another 10% having only one missing attribute. For incident details, four of the five questions are required. The fifth question, on purpose of trip, was optional but 99% complete (Table 1). Most of the collisions and near misses are collisions with moving objects (88%). The object the cyclist collided or nearly collided with was most commonly a vehicle (86%), compared with other objects including cyclists (4%), pedestrians (1%), animals (1%), and infrastructure (9%). Given the high proportion of near miss reporting, it is not surprising that 69% of the incidents did not involve injury. Additionally, 67% of incidents were reported to have occurred while commuting to work or school.

Answering questions on the conditions at the location and time of the collision or near miss is optional but between 80 and 83% were completed (Table 2). Most responses indicate that the roads were dry (65%), sight lines unobstructed (67%), and there were no parked cars (60%). Of those that indicated where they were riding, 43% of riders were on roads with no infrastructure and 73% were heading straight. While the overall report completeness is interesting, data need to be analyzed on a case by case basis to explore the interaction of multiple conditions that are associated with collisions and near misses occurrence.

Personal information on the riders involved in the collision is also optional and only 48–69% complete. Age and sex were the least well reported. Of the 59% of reports that included mapper sex, two-thirds were male, in keeping with typical cyclist profiles for North America. About 68% of citizen mappers ride at least once a week. Most report wearing helmets (67%) and very few report intoxication (1%).

## Discussion

BikeMaps.org is a new global tool for cycling safety data collection. In the future, these data will enable spatial analysis, GIS, and statistics to further knowledge on cycling safety for decision making and planning (19, 20). Our data collection is designed to test hypotheses on infrastructure and traffic flow conditions that lead to injury and safety, and this analysis of early data submissions shows good completeness of attributes. BikeMaps.org will generate more data than has been traditionally available for cycling research and allow quantitative analysis of both *where* and *when* cycling safety varies. With incident data on space and time, across all days of the week, we will be able to assess how safety varies throughout the day with different traffic volumes and flows. For example, in Victoria, Canada where the initial citizen outreach was conducted, Bikemaps.org was able to capture data equivalent to about 1 year of official cycling collision reports within 2 months.

Given the dearth of data available on cycling collisions and near misses, BikeMaps.org is an important tool that can be widely adopted for cycling safety data collection. There are other websites that are also aiming to fill this niche. For instance, collideosco.pe is a cycling incident reporting site for the United Kingdom and Toronto in Canada has adopted an App called Toronto Cycling for collecting of better cycling data. A benefit of BikeMaps.org is the global technology. Technology investments benefit all jurisdictions, and comparisons across locations are more easily made. The primary drawback is that the data collected and displayed need to be consistent. Excellent data available for only one region are difficult to include. When conducting analytics, however, any data can be integrated. In our own research, we anticipate utilizing more detailed ridership and infrastructure data for GIS and spatial analyses.

The near miss data are a substantial contribution for cycling safety research. This gap has been identified in recent studies (3, 12). The benefits of near miss reporting in injury prevention and surveillance are well documented (21–23), including support for early detection of risky locations and increased data. More data will allow statistical modeling to be more robust, assessment of change in safety and risk over space and time, monitoring of change in safety over time. Nearly two-thirds of reports are near misses, which signal the potential to use BikeMaps.org for monitoring.

While it is early for direct comparisons between BikeMaps.org data and official reports, there are several interesting trends that we will continue to monitor. First, while studies have linked 48% of crashes treated at emergency departments have been directly and indirectly related to vehicles (9), 86% of BikeMaps.org data, which includes many less severe incidents, are associated with vehicles. As well, we notice BikeMap.org data are reported in some locations where official reports do not exist. In particular, where biking pathways intersect the road network cyclists are reporting relatively high numbers of incidents.

Beyond data collection BikeMaps.org has mechanisms for promotion of cycling through citizen engagement and increased awareness of cycling safety using mapping as a mechanism. The BikeMaps.org/stats page is an example of a communication tool that can provide positive messaging about how to cycle safely, and may be used to share with citizens the research findings based on the data they contributed. In this way, BikeMaps.org has the potential to narrow the gap between real and perceived cycling risk (24) through better communication.

We are continuing to develop this technology based on feedback and suggestions. In the next phase of development, we are enhancing hazard mapping and real-time alerting of hazards, via text or email, to increase citizen engagement. Hazard mapping provides unique challenges; for example, hazards associated with weather or glass are transient and should not persist on the maps, unless they are associated with prone to the same hazards (e.g., chronic pudding). Infrastructure hazards may be repaired, and a feedback mechanism may be beneficial to remove hazards or update hazard status. Ultimately, this feature can serve as a valuable tool for real-time hazard monitoring, for instance, of road ice or construction.

The observed day of week trends, which indicate most cycling collisions and near misses occur mid-week, are evidence of the need for denominator data in any cycling safety research. Given that most of the mapping is done by commuters (67%), it is not surprising that fewer incidents occur on weekend days. At present, we do not have ridership data to analyze day of week trends though comparison with other research suggests, at a minimum Wednesday and Thursday should have similar levels of ridership [e.g., Ref. (25)]. Rather than only count data, these maps should also show cycling incident risk, for example, as the ratio of incidents relative to the number of riders (1, 18). As a visualization tool on BikeMaps.org, we are utilizing Strava.com data, which shows variation in cycling volumes based on riders that use the Strava App for recording rides. This provides a global backdrop for the website, but further spatial modeling will require more nuanced denominator data.

Citizens are completing the majority of the queried attributes for cycling incidents, but only 48–69% provide personal details. Though all reports are anonymous, citizen mappers seem hesitant to provide personal details. Gender and rider experience have been shown to be important predictors of cycling safety and risk (26). Given these learnings from these early report submissions, we will modify the BikeMaps.org report page to clarify the value of such data, with the hope of more complete demographic data. Specifically, we are adding a “why we ask” button on the data collection form to clarify use of these data and highlight the importance of age and gender details to risk and safety modeling.

Based on consultation with stakeholders, we have many developments planned for BikeMaps.org. We are incorporating new visualizations for the website, such as sliders that allow mappers to visualize incidents that occur over a specific time period. This will allow filtering by time periods, and avoid apparent accumulating risk that is would result from increased reporting. We are also developing a mobile App to encourage timely “on-the-fly” reporting. This will include further functionality, for example, hazard mapping of geotagged photos. Real-time alerts of collisions, near misses, and hazards are also a focus of App development. Alerts, are aimed at letting riders know about short term hazards (e.g., ice, glass, or construction) before they start their rides, allowing for route choice modification for optimal safety. Route mapping will also be included in the App, such that citizens can provide route data on directly through BikeMaps.org. Route choice data can be used to generate the exposure denominator data for incidence risk. Importantly, we need tools for transferring data collected through BikeMaps.org to all researchers and planner in each area, which may be to integrate our website with open data sharing platforms (e.g., Open311) to enable each jurisdiction access to their data.

A forum for collaboration between citizens, advocates, decision makers, and researchers, BikeMaps.org can address a massive data gap that will support safe cycling and increased ridership worldwide. Researchers, advocates, and planners can get involved by encouraging mapping locally. Tools are available to support local outreach activities and, until automated systems are developed, data can be made available upon request.

## Conflict of Interest Statement

The authors declare that the research was conducted in the absence of any commercial or financial relationships that could be construed as a potential conflict of interest.

## Footnotes

^1^http://www.sfcta.org/modeling-and-travel-forecasting/cycletracks-iphone-and-android

^2^http://www.brisksynergies.com

^3^http://www.walkscore.com
